# [Corrigendum] Quercetin-3-methyl ether suppresses human breast cancer stem cell formation by inhibiting the Notch1 and PI3K/Akt signaling pathways

**DOI:** 10.3892/ijmm.2025.5650

**Published:** 2025-09-29

**Authors:** Longbin Cao, Yunxiao Yang, Ziyu Ye, Bihua Lin, Jincheng Zeng, Caihong Li, Tong Liang, Keyuan Zhou, Jixia Li

Int J Mol Med 42: 1625-1636, 2018; DOI: 10.3892/ijmm.2018.3741

Subsequently to the publication of the above article, the authors drew to the Editor's attention that, for the MCF-7 cell invasion assay data shown in Fig. 2C on p. 1630, the '10 *μ*M' data panel had been placed incorrectly, and was a duplication of the data panel shown (correctly) for the '20 *μ*M' experiment. Subsequently, upon performing an independent review of the data published in this paper, the Editorial Office alerted the authors to the fact that, in Fig. 2A, the data shown for the MDA-MB-231 / 20 *μ*M quercetin-3-methyl ester-0 h and -24 h data panels were strikingly similar, such that these were apparently derived from the same original source, where the results of differently performed experiments were intended to have been portrayed.

After re-examining their original data, the authors realized that these data in Fig. 2C had inadvertently been assembled incorrectly. The revised version of Fig. 2, showing the correct data for the MDA-MB-231 / 20 *μ*M quercetin-3-methyl ester-24 h experiment in Fig. 2A and the '10 *μ*M' data panel in Fig. 2C, is shown on on the next page. The authors are grateful to the Editor of *International Journal of Molecular Medicine* for allowing them this opportunity to publish a Corrigendum, and all the authors agree with its publication. Furthermore, the authors apologize to the readership for any inconvenience caused.

## Figures and Tables

**Figure 2:**
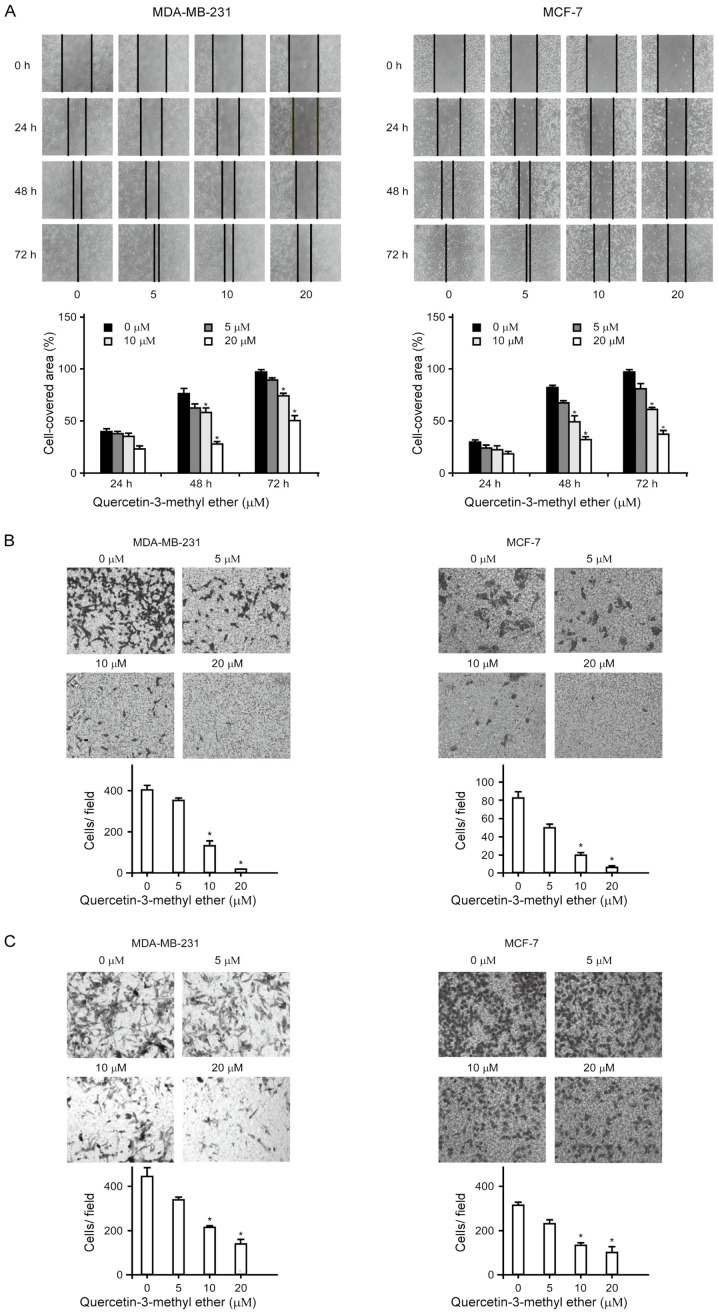
Quercetin-3-methyl ether significantly inhibits the migration and invasion of breast cancer cells. (A) Quercein-3-methyl ether inhibited cell motility. Cells were treated with 0–20 *μ*M quercetin-3-methyl ether for 24–72 h. Cell motility was determined using a scratch wound-healing assay. Images were captured using a microscope (magnification, ×100). Closure rate is shown as a percentage of wound closure. Data are presented as the mean ± SD. ^*^P<0.05 quercetin3-methyl ether, vs. DMSO. (B) Quercein-3-methyl ether inhibited cell migration. Cells were treated with 0–20 *μ*M quercetin-3-methyl ether for 48 h. Cell migration was examined using a Transwell assay. Cells that penetrated the lower compartment were stained with crystal violet. The cell numbers were counted under an inverted microscope (magnification, ×100). Data are presented as the mean ± SD. ^*^P<0.05 quercetin3-methyl ether, vs. DMSO. (C) Quercein-3-methyl ether inhibited cell invasion. Cells were treated with 0–20 *μ*M quercetin-3-methyl ether for 48 h. Cell invasion was examined using the Matrigel Transwell chamber assay. Cells that invaded the lower chamber were stained with crystal violet. The cell numbers were counted under an inverted microscope (magnification, ×100). Data are presented as the mean ± SD. ^*^P<0.05 quercetin3-methyl ether, vs. DMSO. SD, standard deviation.

